# DCAMCP: A deep learning model based on capsule network and attention mechanism for molecular carcinogenicity prediction

**DOI:** 10.1111/jcmm.17889

**Published:** 2023-07-31

**Authors:** Zhe Chen, Li Zhang, Jianqiang Sun, Rui Meng, Shuaidong Yin, Qi Zhao

**Affiliations:** ^1^ School of Mathematics and Statistics Liaoning University Shenyang China; ^2^ School of Life Science Liaoning University Shenyang China; ^3^ School of Information Science and Engineering Linyi University Linyi China; ^4^ School of Computer Science and Software Engineering University of Science and Technology Liaoning Anshan China

**Keywords:** capsule network, carcinogenicity, computational toxicology, deep learning, graph attention network

## Abstract

The carcinogenicity of drugs can have a serious impact on human health, so carcinogenicity testing of new compounds is very necessary before being put on the market. Currently, many methods have been used to predict the carcinogenicity of compounds. However, most methods have limited predictive power and there is still much room for improvement. In this study, we construct a deep learning model based on capsule network and attention mechanism named DCAMCP to discriminate between carcinogenic and non‐carcinogenic compounds. We train the DCAMCP on a dataset containing 1564 different compounds through their molecular fingerprints and molecular graph features. The trained model is validated by fivefold cross‐validation and external validation. DCAMCP achieves an average accuracy (ACC) of 0.718 ± 0.009, sensitivity (SE) of 0.721 ± 0.006, specificity (SP) of 0.715 ± 0.014 and area under the receiver‐operating characteristic curve (AUC) of 0.793 ± 0.012. Meanwhile, comparable results can be achieved on an external validation dataset containing 100 compounds, with an ACC of 0.750, SE of 0.778, SP of 0.727 and AUC of 0.811, which demonstrate the reliability of DCAMCP. The results indicate that our model has made progress in cancer risk assessment and could be used as an efficient tool in drug design.

## 1 INTRODUCTION

Cancer is one of the leading causes of death in the world. There are various causes of cancer, and the survey shows that the most important cause is the presence of carcinogens in food, tobacco and beverages.^1^, ^2^ Therefore, we must pay attention to great importance to these carcinogens. Any substance that can induce tumours, increase the incidence of tumours or shorten the time to tumorigenesis is defined as a carcinogen. Carcinogens can increase tumour incidence by directly interacting with DNA or disrupting cellular metabolic processes.^3^ Every day, a large number of synthetic chemicals are manufactured to meet demand, and during these synthetic processes, the chemical properties of the molecules may be transformed due to changes in the molecular structure, leading to the formation of carcinogens. As a result, the carcinogenicity assessment of these new compounds is very necessary. Carcinogenicity prediction and cancer risk assessment are critical not only for regulatory purposes, but also for drug discovery and development. In general, most of our knowledge about carcinogens is derived from data related to carcinogenicity studies in rodents.^4^ However, these animal experiments are not only time‐consuming and labor‐intensive, but even unethical. Therefore, the use of computational models to predict the carcinogenicity of compounds based on structural information has been recognized as a less costly ancillary solution and has recently become the focus of research.^5^, ^6^, ^7^

During recent years, many methods for predicting the carcinogenicity of compounds have been developed in previous studies. These methods are broadly classified as qualitative structure–activity relationship (SAR) models,^8^, ^9^, ^10^, ^11^, ^12^ that is, classification models, quantitative structure–activity relationship (QSAR) models^13^, ^14^, ^15^ and expert systems.^16^, ^17^, ^18^ In 2010, Fjodorova et al. used the back‐propagation artificial neural network technique to create a quantitative model. On the test set, their model performed well in terms of prediction, which achieved accuracy of 68%, sensitivity of 73% and specificity of 63%.^19^ After that, Singh et al. developed models of probabilistic neural networks and generalized regression neural networks to differentiate carcinogens.^20^ At the same time, Tanabe et al. proposed a novel sensitivity analysis method for variable selection in support vector machines (SVMs) to improve the performance level of QSAR models for predicting carcinogenicity.^11^ Besides, in 2013, Zhong et al. developed a SVM‐based classification model that allows the classification of carcinogenicity of non‐homogeneous chemicals.^21^ In 2016, Zhang et al. constructed a naive Bayes classifier with an accuracy of 68 ± 1.9% on the external validation set.^22^ In 2017, Zhang et al. proposed a set of ensemble models for classification, the best of which was the Ensemble XGBoost model, which achieved 70% accuracy on the external validation set.^23^ Later, in 2019, Wang et al. proposed a deep learning model named CapsCarcino, which achieved 85% accuracy, 82.6% sensitivity and 88.2% specificity on the external validation set.^24^ In 2022, Fradkin et al. used a graph neural network for the first time to identify carcinogenic molecules and achieved good performance.^25^ Despite the fact that these methods have demonstrated reasonable predictive power, there is still a lot of room for experimentation and advancement in the use of deep learning algorithms in this field.

Deep learning is a relatively new research area in the field of machine learning. It has been used in a variety of applications, including miRNA‐disease associations prediction,^26^, ^27^, ^28^ metabolite‐disease associations prediction,^29^ lncRNA‐miRNA interactions prediction^30^, ^31^, ^32^ and circRNA‐disease associations prediction.^33^, ^34^ In 2017, Hinton et al. proposed a new deep learning architecture called Capsule Network (CapsNet).^35^ Unlike traditional neurons, the input and output of CapsNet are vectors. The length of the vector can be understood as the probability in traditional neurons, and the direction of the vector as the representation of other information, for example, position information. CapsNet has been used successfully in a variety of fields, most notably image processing. Wang et al. were the first to propose the use of capsule networks in drug discovery and design, and they successfully demonstrated their effectiveness in predicting carcinogens and non‐carcinogens.^24^ As the capsule network is still in its early stage, many researchers have conducted extensive research on its effectiveness. Mazzia et al. developed a non‐iterative, highly parallelisable self‐attention routing algorithm.^36^ This is an improvement on the internal algorithm of the capsule network. Compared with the original dynamic routing, self‐attention routing greatly reduces the number of trainable parameters and improves the generalisation ability.^36^ This also provides an inspiration for our research in this study. In addition, molecular graph representation learning has gradually become a hot spot in the field of toxicity prediction. As the expression of molecules in most previous studies is limited to molecular fingerprints and molecular descriptors, the study of their graph characteristics is also worth exploring.

In this work, we build a deep learning model based on capsule network and attention mechanism called DCAMCP. To characterize molecules more comprehensively, we not only provide 12 different molecular fingerprints of the molecules, but also generate respective graph features for them. We further employ two feature selection methods to remove redundant features in molecular fingerprints to make the features more reliable. Similarly, we extract reliable features from molecular graph features by building graph attention neural network layers. Furthermore, we build a classification network based on self‐attention routing capsule network to make predictions. We assess the performance of DCAMCP in fivefold cross‐validation experiment and the results outperform most of the existing methods. Moreover, DCAMCP also achieves excellent results on the external validation set, which shows the reliability and robustness of DCAMCP. These results suggest that DCAMCP is a valid and feasible model for carcinogenicity risk assessment.

## 2 MATERIALS AND METHODS

### 2.1 Data preparation

Currently, animal experiments are our primary source of information on carcinogenic compounds, and several on‐line databases of rodent carcinogenicity are available. The datasets that we use to develop our model are selected from three of these databases, and their detailed descriptions are as follows:

1. CPDB database is a single standardized resource that contains the results of chronic, long‐term animal cancer tests conducted since the 1950s, providing 1547 chemicals from 429 NCI/NTP (National Cancer Institute/National Toxicology Program) technical reports. These compounds data are primarily based on experiments in mice, rats and hamsters.^37^ (https://www.nlm.nih.gov/databases/download/cpdb.html)
2. CCRIS database contains chemical records with carcinogenicity, mutagenicity, tumour promotion and tumour inhibition test results. It is developed by the NCI (National Cancer Institute). This database provides carcinogenicity data for more than 4500 compounds based on rodent (rat, mouse) experiments. Test results have been reviewed by experts in carcinogenesis and mutagenesis. (https://www.nlm.nih.gov/databases/download/ccris.html)
3. ISSCAN database is curated by the Istituto Superiore di Sanità and contains long‐term carcinogenicity bioassay results on rodents (rat, mouse). The carcinogenic results have been critically reviewed.^38^ (http://www.iss.it/ampp/dati/cont.php?id=233&lang=1&tipo=7)

We prioritize carcinogenicity data based on rat experiments in the above three databases; because, the results of rat experiments are thought to be more suitable for predicting human carcinogenicity.^39^, ^40^ In order to build a reliable predictive model, we must exclude the following compounds: (1) compounds containing less than three carbon atoms, which have simple structures that do not provide sufficient training characteristics; (2) compounds containing heavy metals that significantly affect their toxicity; (3) polymers, because the characterisation method we use is not suitable for feature extraction of polymers; (4) mixtures, the reason is that the mixtures contain multiple compounds and it is not possible to determine which compound is carcinogenic. Finally, we choose 1003 compounds from the CPDB database (494 carcinogens and 509 non‐carcinogens), 927 compounds from the CCRIS database (429 carcinogens and 498 non‐carcinogens) and 40 compounds from the ISSCAN database (23 carcinogens and 17 non‐carcinogens). After deduplication, there are totally 1664 different compounds obtained.

**FIGURE 1:**
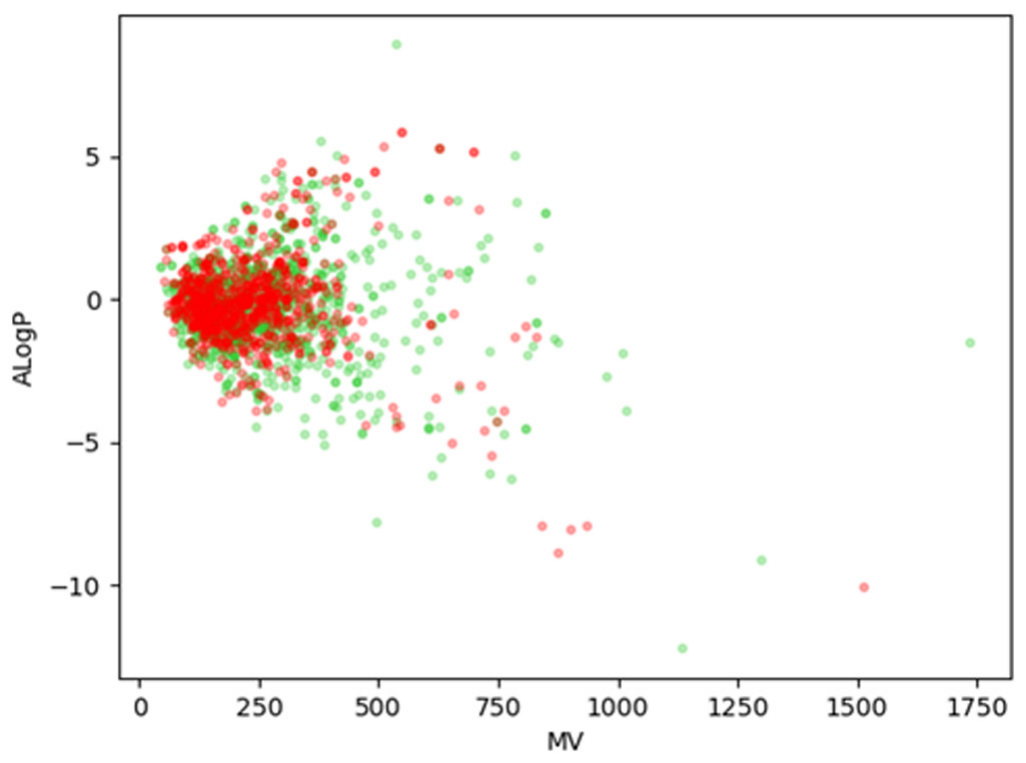
Chemical space of the training set. The chemical space is defined by the molecular weight (MW) on the X‐axis and the logarithm of the octanol/water partition coefficient (ALogP) on the Y‐axis. Carcinogens and non‐carcinogens are represented by red and green dots, respectively.

Because of the limited size of the dataset, we attempt to make our model as fully trained as possible. We try to divide out different training sets to train DCAMCP. When the number of training sets is less than 1500 molecules, the training effect of DCAMCP improves with the increase of the number of molecules. When the number of molecules is raised from 1500 to 1600, there is no significant improvement in the performance of DCAMCP. As a result, we decide to randomly use 1564 of these compounds as the training set (726 carcinogens and 838 non‐carcinogens) and the remaining 100 compounds as the external validation set (45 carcinogens and 55 non‐carcinogens). It is well known that the diversity of compounds in the database has a significant impact on the predictive accuracy of the model. To ensure that a reliable model can be developed, we therefore illustrate the chemical spatial distribution of the training set by scatterplots of molecular weight (MW) versus log of the octanol/water partition coefficient (AlogP) for both carcinogens and non‐carcinogens. As shown in Figure 1, the MW and ALogP distributions of carcinogenic and non‐carcinogenic compounds are similar, with a MW range of between 50 and 1000 Da and AlogP range of between −5 and 5, which is a broader range than that of most compounds. This implies that the datasets have similar chemical spaces with good distributional consistency, which is critical for developing a stable predictive model. It is also clear from Figure 1 that we cannot use MW and ALogP alone to distinguish between carcinogenic and non‐carcinogenic compounds.

### 2.2 Architecture of DCAMCP

**FIGURE 2:**
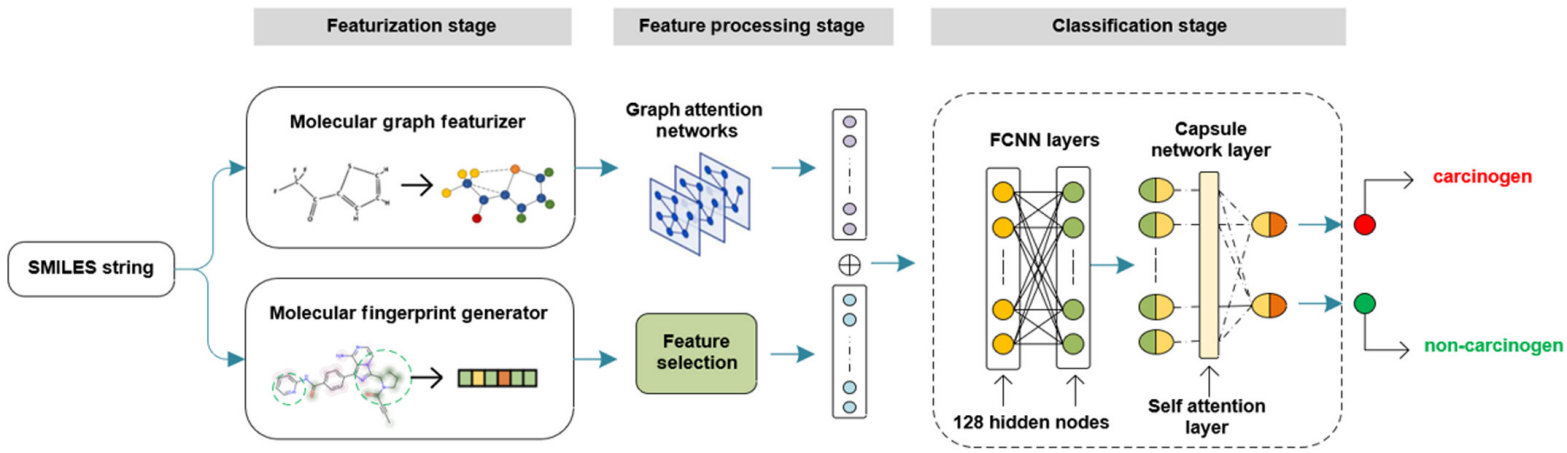
The workflow of DCAMCP.

The framework of DCAMCP is shown in Figure 2. As one can see from the flowchart, DCAMCP is divided into three parts, which are termed as featurisation stage, feature processing stage and classification stage. Briefly, we first generate both molecular fingerprints and molecular graph features for each molecule in order to characterize them. Then, we use two feature selection methods and graph attention neural network layers to process these two features, respectively. Finally, we construct a classification network consisting of two fully connected layers and a capsule network layer to make classification predictions on the processed features. The specifics of these three parts are provided below.

### 2.3 Featurisation stage

To train DCAMCP to recognize molecular structures, we must characterize molecules themselves at first. At this stage, we generate two features for each molecule: molecular fingerprints and molecular graph features. The details and implementation of these two features are as follows.

#### 2.3.1 Molecular fingerprints

Molecular fingerprints are abstract representations of molecules. They map molecular structure into a set of numbers or binary values that reflect whether a specific molecule substructure exists in a molecule, and these specific structures are thought to play important roles in explaining molecular activities. Most compound toxicity predictive models have employed molecular fingerprints to represent molecules. Currently, many different forms of molecular fingerprints can be generated in various software. In order to better enable our model to identify the structural features of molecules, we use the following 12 molecular fingerprints in this study: MDL Molecular ACCess fingerprint (MACCS, 166 bits), PubChem fingerprint (PubChem), CDK fingerprint (CDK), CDK extended fingerprint (CDKExt), Klekota‐Roth fingerprint (KR), 2D atom pairs fingerprint (AP2D), Klekota‐Roth count fingerprint (KRC), 2D atom pairs count fingerprint (AP2DC), Substructure fingerprint (FP4), Substructure count fingerprint (FP4C) and Estate fingerprint (Estate). All fingerprints are calculated by PaDEL‐Descriptor software (version 2.21).^41^ The names, types and lengths of these fingerprints are summarized in Table 1.

**TABLE 1:** Summary of 12 types of molecular fingerprints.

Fingerprint name	Abbreviation	Type	Size (bits)	Selected (bits)
CDK	CDK	Hash fingerprints	1024	929
CDK extended	CDKExt	Hash fingerprints	1024	944
CDK graph	CDKGraph	Hash fingerprints	1024	225
MACCS	MACCS	Structural features	166	83
PubChem	PubChem	Structural features	881	104
Klekota‐Roth	KR	Structural features	4860	100
Klekota‐Roth count	KRC	Structural features count	4860	155
2D atom pairs	AP2D	Structural features	780	42
2D atom pairs count	AP2DC	Structural features count	780	81
Substructure	FP4	Structural features	307	32
Substructure count	FP4C	Structural features count	307	43
Estate	Estate	Structural features	79	19

#### 2.3.2 Molecular graph features

Molecular graph features are simply to extract and compress the features of compound structure graphs, turn all molecular models into graphs and use appropriate topological indices to encode the graph structure. Molecular graph features can convey molecular topological information in an intuitive and concise manner. As some molecules may not be distinguished solely by fingerprints, it is also vital to evaluate the graph features of molecules. Specifically, we characterize the molecules by using functions from the DGL‐Life algorithm library.^42^ DGL‐Life is a graph neural network algorithm library for the chemical and biological fields. We use the SMILES string of the molecule as input to characterize the atoms of the molecule through the CanonicalAtomFeaturizer function. After that, we similarly characterize the bonds connecting the atoms with the CanonicalBondFeaturizer function. Finally, we use the smiles_to_bigraph function to generate full graph features for each molecule.

### 2.4 Feature processing stage

In order to express the molecular structure more finely, we need to process the features. At this stage, we use the feature selection method to filter out the useless features in molecular fingerprints, and use the graph attention network layer to learn molecular graph features that are more conducive to prediction. The specific details and implementation methods are as follows.

#### 2.4.1 Feature selection

As some bits of molecular fingerprints may not be helpful for predictive results, we need remove them through feature selection. Feature selection is a critical step in data processing, it can be used to eliminate irrelevant or redundant features, reducing the number of features, improving model accuracy and decreasing running time. To filter features in this study, two methods are used: low‐variance feature filtering and high‐correlation feature filtering. Among them, low‐variance feature filtering aims to remove some features whose variance is approximately zero (variance <0.05), that is, features that have the same value in most samples. The variance *D(X)* is calculated as follows:

(1) $$D\left(X\right)=E\left(X^{2}\right)-E^{2}\left(X\right)$$

where *E(X)* is the expectation of X. Whereas high‐correlation feature filtering aims to delete some features that are overly correlated with each other (Pearson's correlation coefficients >0.7), and keep only one of these highly correlated features. The formula for Pearson's correlation coefficient is:

(2) $$\rho_{X,Y}=\frac{\mathrm{cov}\left(X,Y\right)}{\sigma_{X}\sigma_{Y}}=\frac{E\left(\mathrm{XY}\right)-E\left(X\right)E\left(Y\right)}{\sqrt{E\left(X^{2}\right)-E^{2}\left(X\right)}\sqrt{E\left(Y^{2}\right)-E^{2}\left(Y\right)}}$$

where cov is the covariance and σ is the standard deviation. Specifically, we implement the above two works through the *variancethreshold* function in the sklearn algorithm library and the custom *findcorrelated* function.

#### 2.4.2 Graph attention network

Graph attention network (GAT) is a novel neural network architecture proposed in recent years that operates on graph‐structured data, which utilizes masked self‐attention layers to address the shortcomings of previous methods based on graph convolutions or their approximations.^43^ Due to its effectiveness on graph feature processing, in this study, we adopt it to process our molecular graph features. The following are the core formulas of a graph attention network layer:

(3) $${Z_{i}}^{\left(l\right)}=W^{\left(l\right)}{H_{i}}^{\left(l\right)}$$

(4) $$e_{\mathrm{ij}}=\text{LeakyReLu}\left({\vec{a^{\left(l\right)}}}^{T}\left({z_{i}}^{\left(l\right)}\right)\|{z_{j}}^{\left(l\right)}\right)$$

(5) $${a_{\mathrm{ij}}}^{\left(l\right)}={\text{softmax}}_{j}\left(e_{\mathrm{ij}}\right)=\frac{\exp\left({e_{\mathrm{ij}}}^{\left(l\right)}\right)}{\sum_{k\in N\left(i\right)}\exp\left({e_{\mathrm{ik}}}^{\left(l\right)}\right)}$$

(6) $${H_{i}}^{\left(l+1\right)}=\sigma \left[\sum_{j\in N\left(i\right)}{a_{\mathrm{ij}}}^{\left(l\right)}{z_{j}}^{\left(l\right)}\right]$$

These formulas represent the operation process of node *i* in a graph attention network layer. Among them, $H_{i}^{\left(l\right)}$ represents the vector feature of the node *i* as the input of the graph attention network layer, and $H_{i}^{\left(l+1\right)}$ is its output, meaning the new vector feature of the node *i*. Equations (3) and (6) denote the relationship between $H_{i}^{\left(l\right)}$ and $H_{i}^{\left(l+1\right)}$, where $W^{\left(l\right)}$ is the weight matrix, $Z_{i}^{\left(l\right)}$ is the vector feature of node *i* after linear transformation and $a_{\mathrm{ij}}^{\left(l\right)}$ is the attention parameter between nodes. $a_{\mathrm{ij}}^{\left(l\right)}$ is generated by Equations (4) and (5). The attention weight $e_{\mathrm{ij}}$ between two nodes is calculated by Equation (4), and its calculation process is similar to a single‐layer feedforward neural network, using LeakyReLu as the activation function. Then, to make $a_{\mathrm{ij}}^{\left(l\right)}$ easier to calculate and compare, we normalize $e_{\mathrm{ij}}$ through the softmax layer and calculate the final $a_{\mathrm{ij}}^{\left(l\right)}$ by Equation (5). Furthermore, two layers of graph attention network are employed to extract graph features in our study. We finally generate its respective graph feature vector for each molecule through the MaxPooling function.

### 2.5 Classification stage

After getting the molecular features, we next build a classification network. At this stage, we introduce the composition and operation of the classification network in detail. First, the classification network consists of two fully connected layers and a capsule network layer, where the capsule network layer uses a self‐attention routing algorithm for internal transfer operations. The output units of the two fully connected layers are both set to 128, and the batch normalisation layer and the dropout layer are added after the output of each fully connected layer. Second, we get a 128‐dimensional vector after going through the fully connected layer. For smooth input to the capsule network layer, we pack it into 16 capsules and each capsule contains an 8‐dimensional vector. Following a series of operations at the capsule network layer we generate 2 capsules, each of which contains a 2‐dimensional vector. The outputs of the capsule network layer are predicted classification labels corresponding to carcinogens and non‐carcinogens. As the algorithm of the fully connected layer has been described in detail in the previous literature,^44^ we only introduce the self‐attention routing algorithm of the capsule network layer below.

#### 2.5.1 Algorithm of capsule network

The capsule network is a network based on multidimensional vector operations. Despite the extra dimension, the overall architecture is very similar to a fully connected network, The self‐attention routing algorithm adds a layer of self‐attention layer to the original algorithm and its calculation formula with the input layer is as follows:

(7) $${\hat{U}}_{\left(n^{l},n^{l+1},d^{l+1}\right)}^{l}=u_{n}^{\mathrm{Tl}}\times W_{\left(n^{l},n^{l+1},:\right)}^{l}$$

where *l* represents the number of layers, *n* means the number of capsules and *d* denotes the dimension of capsules. $W_{\left(n^{l},n^{l+1},:\right)}^{l}$ is the weight matrix of the *l*‐*th* layer. The vectors in each capsule are linearly transformed by multiplying with $W_{\left(n^{l},n^{l+1},:\right)}^{l}$ to generate ${\hat{U}}_{\left(n^{l},n^{l+1},d^{l+1}\right)}^{l}$. Then, the calculation formula between the self‐attention layer and the output layer is,

(8) $$s_{n}^{l+1}={\hat{U}}_{\left(:,n^{l+1},:\right)}^{\mathrm{Tl}}\times \left(C_{\left(:,n^{l+1}\right)}^{l}+B_{\left(:,n^{l+1}\right)}^{l}\right)$$

where $B_{\left(:,n^{l+1}\right)}^{l}$ is the log priors matrix containing all weights. On the other hand, $C_{\left(n^{l},n^{l+1}\right)}^{l}$ is the matrix containing all coupling coefficients produced by the self‐attention algorithm. The formula for generating self‐attention matrix $C_{\left(n^{l},n^{l+1}\right)}^{l}$ is,

(9) $$A_{\left(:,:,n^{l+1}\right)}^{l}=\frac{{\hat{U}}_{\left(:,n^{l+1},:\right)}^{l}\times {\hat{U}}_{\left(:,n^{l+1},:\right)}^{\mathrm{Tl}}}{\sqrt{d^{l}}}$$

(10) $$C_{\left(:,n^{l+1}\right)}^{l}=\frac{\exp\left(\sum_{n^{l}}A_{\left(:,n^{l},n^{l+1}\right)}^{l}\right)}{\sum_{n^{l+1}}\exp\left(\sum_{n^{l}}A_{\left(:,n^{l},n^{l+1}\right)}^{l}\right)}$$

which contains a symmetric matrix $A_{\left(:,:,n^{l+1}\right)}^{l}$ generated by multiplying matrix ${\hat{U}}_{\left(n^{l},n^{l+1},d^{l+1}\right)}^{l}$ with its own transpose. *d* is a hyperparameter for stable training, helping to maintain a balance between coupling coefficients and log priors. Finally, $A_{\left(:,:,n^{l+1}\right)}^{l}$ generates the coupling coefficient matrix $C_{\left(n^{l},n^{l+1}\right)}^{l}$ through the calculation of the softmax layer. Besides, the initial input and final output of the capsule network are calculated by the activation function, which is shown in the following formula:

(11) $$v_{n}^{l}=\text{squash}\left(s_{n}^{l}\right)=\left(1-\frac{1}{e^{\left\|s_{n}^{l}\right\|}}\right)\frac{s_{n}^{l}}{\left\|s_{n}^{l}\right\|}$$

For the output of the capsule layer, we count its loss function $L_{k}$ by the formula as,

(12) $$L_{k}=T_{k}\max{\left(0,m^{+}-\left\|v_{k}\right\|\right)}^{2}+\lambda \left(1-T_{k}\right)\max{\left(0,\left\|v_{k}\right\|-m^{-}\right)}^{2}$$

where $T_{k}=1$ if the predicted value is equal to the real value and $T_{k}=0$ otherwise. We set $m^{+}$ to 0.9, $m^{-}$ to 0.1 and λ to 0.5, which have been shown to ensure the stability of the training process.^35^, ^36^ Furthermore, we use Adam optimizer to update all learnable parameters in our model through gradient descent algorithm. Besides, we give detailed hyperparameters for each part of DCAMCP and list them in Table 2.

**TABLE 2:** Hyperparameter settings for DCAMCP.

Structure	Hyper‐parameters	Optimum values
Graph attention network layer	Hidden‐feats	50
	Num‐heads	4
	Feat‐drops	0.2
	Attn‐drops	0.2
	Alphas	0.2
Capsule network layer	Number of input capsules	16
	Dim of input capsules	8
	Number of output capsules	2
	Dim of output capsules	2
	Parameter D	128
Fully connected layer	Number of units	128
	Dropout	0.2
Training parameters	Batch size	256
	Learning rate	0.001
	Weight decay	0.001
	Epoch	100

## 3 RESULTS

### 3.1 Performance evaluation

To validate the performance of DCAMCP, we evaluate it using fivefold cross‐validation (fivefold CV) with 100 repetitions and external validation. In fivefold CV we split the original sample into five equal subsamples. Four of the five subsamples are used as training data, and the remaining one is used as validation data to test DCAMCP. This process is then repeated five times, with each of the five subsamples being used as validation data only once. Furthermore, repeating the entire process 100 times is intended to reduce randomness in the results and provide a robust performance evaluation. The following metrics are used to evaluate the predictive performance of DCAMCP, including accuracy (ACC, overall predictive accuracy), sensitivity (SE, predictive accuracy for carcinogens), specificity (SP, predictive accuracy for non‐carcinogens) and the area under the receiver‐operating characteristic curve (AUC). The corresponding formulas are as follows:

(13) $$\mathrm{SE}=\frac{\mathrm{TP}}{\mathrm{TP}+\mathrm{FN}}$$

(14) $$\mathrm{SP}=\frac{\mathrm{TN}}{\mathrm{TN}+\mathrm{FP}}$$

(15) $$\mathrm{ACC}=\frac{\mathrm{TP}+\mathrm{TN}}{\mathrm{TP}+\mathrm{TN}+\mathrm{FN}+\mathrm{FP}}$$

where TP (true positive) is the number of carcinogens correctly predicted, TN (true negative) is the number of non‐carcinogens correctly predicted, FP (false positive) is the number of non‐carcinogens incorrectly predicted as carcinogens and FN (false negative) is the number of carcinogens mispredicted as non‐carcinogens.

The receiver operating characteristic curve (ROC) is a plot of the TP rate (sensitivity) against the FP rate (1‐specificity) for the different possible threshold points of a test. The AUC is the area under the ROC curve, which is an important indicator of classifiers.

### 3.2 Comparison with other methods

In this subsection, we investigate the performance of DCAMCP by fivefold CV and rigorously assess the ability of DCAMCP to discriminate carcinogens. In order to better train DCAMCP, we generate twelve molecular fingerprints and respective molecular graph features for all 838 non‐carcinogens and 726 carcinogens in the dataset. The performance of DCAMCP is evaluated through fivefold CV and external validation sets using four evaluation metrics (ACC, SE, SP and AUC).

As the molecular graph features are generated in the same manner, we mainly focus on the results obtained for various molecular fingerprints. As a result, we present the performance of different molecular fingerprints in Table 3. It can be seen that DCAMCP performs the best on the CDKExt fingerprint, with an ACC of 0.718, SE of 0.721, SP of 0.715 and AUC of 0.793.

**TABLE 3:** Performance of DCAMCP on the training dataset under 12 molecular fingerprints in fivefold CV.

Fingerprints	ACC	SE	SP	AUC
CDK	0.712 ± 0.008	0.710 ± 0.013	0.713 ± 0.016	0.788 ± 0.015
CDKExt	**0.718 ± 0.009**	**0.721 ± 0.006**	0.715 ± 0.014	**0.793 ± 0.012**
CDKGraph	0.692 ± 0.012	0.669 ± 0.032	0.711 ± 0.034	0.734 ± 0.022
MACCS	0.676 ± 0.012	0.649 ± 0.025	0.701 ± 0.032	0.722 ± 0.016
PubChem	0.685 ± 0.013	0.656 ± 0.032	0.709 ± 0.023	0.737 ± 0.024
KR	0.685 ± 0.011	0.686 ± 0.026	0.684 ± 0.024	0.736 ± 0.013
KRC	0.692 ± 0.014	0.713 ± 0.018	0.673 ± 0.021	0.750 ± 0.017
AP2D	0.666 ± 0.012	0.643 ± 0.019	0.684 ± 0.027	0.680 ± 0.023
AP2DC	0.630 ± 0.014	0.536 ± 0.022	0.706 ± 0.023	0.658 ± 0.024
FP4	0.589 ± 0.013	0.367 ± 0.023	0.800 ± 0.024	0.668 ± 0.021
FP4C	0.627 ± 0.016	0.365 ± 0.026	**0.855 ± 0.018**	0.680 ± 0.021
Estate	0.632 ± 0.013	0.612 ± 0.023	0.651 ± 0.021	0.661 ± 0.031

The significance for bold values highlight the maximum value in each column.

Because most previous methods are not publicly available, we cannot compare with them directly. Although we reproduce the previous state‐of‐the‐art model which is CapsCarcino, we only achieve an ACC of 0.706 and an AUC of 0.769, which is far from the results of the original paper. Meanwhile, we try to contact the authors, but get no response. As a result, to illustrate the effectiveness of our method, we compare DCAMCP with five common machine learning algorithms including KNN, SVM, XGBoost, RF and DNN on the same dataset. We adopt CDKExt fingerprint as a feature and present the results of these methods in Table 4. It can be seen that the AUC of DCAMCP is 0.793, which is 6.7%, 1.5%, 0.9%, 3.6% and 3.1% higher than that of KNN, SVM, XGBoost, RF and DNN, respectively. Among these five methods, DNN achieves relatively high SE of 0.704 and ACC of 0.712, which are 1.7% and 0.6% lower than those of DCAMCP, respectively. XGBoost achieves a relatively high SP of 0.834, which is 11.9% higher than that of DCAMCP. Although SVM and XGBoost perform better on the SP metric, they are not as good as DCAMCP in the other three indicators. In short, DCAMCP achieves the best performances on SE, ACC and AUC metrics. These results suggest that DCAMCP is superior to other methods in carcinogenicity prediction.

**TABLE 4:** Performance of DCAMCP and five common machine learning models in fivefold CV under the same training dataset.

Model	SE	SP	ACC	AUC
DCAMCP	**0.721 ± 0.006**	0.715 ± 0.014	**0.718 ± 0.009**	**0.793 ± 0.012**
KNN	0.674 ± 0.026	0.661 ± 0.027	0.667 ± 0.032	0.726 ± 0.036
SVM	0.644 ± 0.031	0.764 ± 0.028	0.708 ± 0.036	0.778 ± 0.028
XGBoost	0.566 ± 0.038	**0.834 ± 0.027**	0.709 ± 0.036	0.784 ± 0.029
RF	0.675 ± 0.026	0.699 ± 0.028	0.688 ± 0.035	0.757 ± 0.035
DNN	0.704 ± 0.008	0.719 ± 0.011	0.712 ± 0.012	0.762 ± 0.021

The significance for bold values highlight the maximum value in each column.

### 3.3 External validation

To demonstrate the reliability of DCAMCP, we use an external validation set of 100 compounds to test it. As these compounds are not involved in the construction of DCAMCP, the resulting performance reflects the ability of our model to predict the carcinogenicity of new compounds. The predictive performance of molecular fingerprints on the external validation set is shown in Table 5, according to which the performance of DCAMCP on the external validation set under different molecular fingerprints is similar to that in Table 3, and CDKExt fingerprint still achieves the best results, with ACC of 0.750, SE of 0.778, SP of 0.727 and AUC of 0.811. These results show that the performance of DCAMCP on the external validation set is also excellent. Furthermore, we present the performance of the above‐mentioned five machine learning models on this external validation set in Table 6. We can see that the AUC of KNN, SVM, XGBoost, RF and DNN are 0.730, 0.752, 0.788, 0.741 and 0.788. Furthermore, they are 8.1%, 5.9%, 2.3%, 7% and 2.3% lower than that of DCAMCP, respectively. Among these five methods, DNN achieves relatively high ACC of 0.74 and SE of 0.778. Compared with DCAMCP, its SE is the same as DCAMCP, but its ACC is lower by 1%. Although XGBoost achieves a relatively high SP of 0.83 which is 10.3% higher than that of DCAMCP. It can be seen that DCAMCP still achieves the best performances on SE, ACC and AUC metrics. The above results indicate that DCAMCP has stronger generalisation ability compared with other methods.

**TABLE 5:** Performance of DCAMCP on the external validation.

Fingerprints	ACC	SE	SP	AUC
CDK	0.730	0.756	0.709	0.786
CDKExt	**0.750**	**0.778**	0.727	**0.811**
CDKGraph	0.680	0.756	0.618	0.755
MACCS	0.670	0.756	0.600	0.715
PubChem	0.640	0.644	0.636	0.708
KR	0.690	0.667	0.709	0.728
KRC	0.680	0.756	0.618	0.728
AP2D	0.640	0.644	0.636	0.666
AP2DC	0.580	0.467	0.673	0.590
FP4	0.610	0.556	0.655	0.583
FP4C	0.590	0.400	**0.745**	0.652
Estate	0.590	0.578	0.600	0.570

The significance for bold values highlight the maximum value in each column.

**TABLE 6:** Performance of DCAMCP and five common machine learning models on the same external validation dataset.

Model	SE	SP	ACC	AUC
DCAMCP	**0.778**	0.727	**0.750**	**0.811**
KNN	0.688	0.675	0.680	0.730
SVM	0.673	0.715	0.700	0.752
XGBoost	0.606	**0.830**	0.730	0.788
RF	0.689	0.649	0.670	0.741
DNN	0.778	0.708	0.740	0.788

The significance for bold values highlight the maximum value in each column.

### 3.4 Ablation experiments

In order to ensure that each component in DCAMCP can positively contribute to the predictive results, we perform ablation experiments and validate the performance of our model on the external validation set after removing different components.

1. DCAMCP‐NA: It employs a dynamic routing algorithm that eliminates the attention mechanism of capsule networks to predict the carcinogenicity of molecules.
2. DCAMCP‐OF: It uses only CDKExt fingerprint features as input to predict the carcinogenicity of molecules.
3. DCAMCP‐OG: It applies only molecular graph features as input to predict the carcinogenicity of molecules.

**FIGURE 3:**
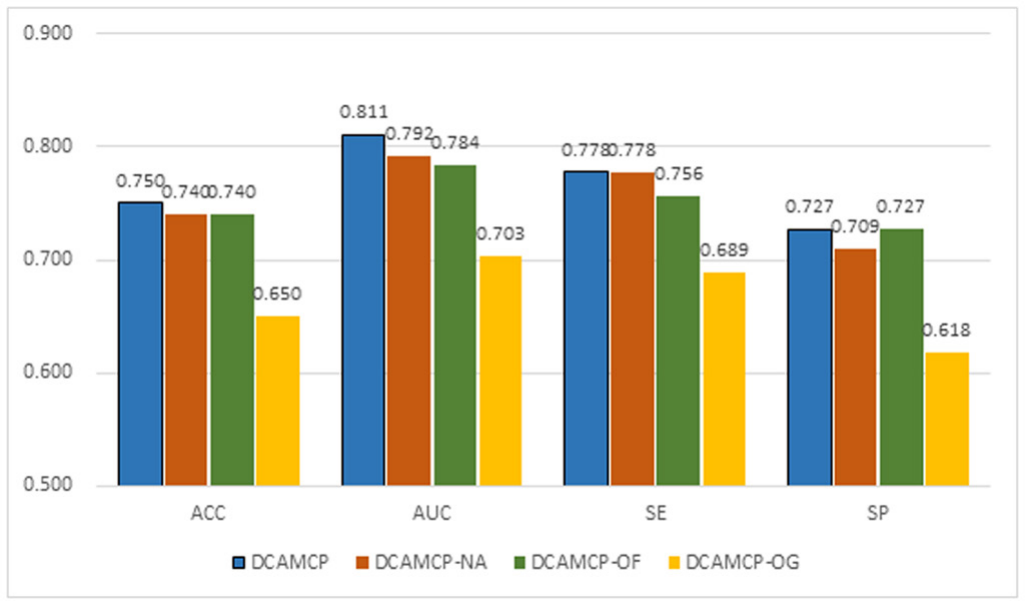
Comparison analysis between DCAMCP and its ablation experiments on the external validation.

We show the performance of these models through the bar plot in Figure 3. It can be seen that DCAMCP‐OF has an ACC of 0.74, SE of 0.756, SP of 0.727 and AUC of 0.784. Compared with DCAMCP, its SP is the same as DCAMCP, but its ACC, SE and AUC are lower by 1%, 2.2% and 2.7%, respectively. And DCAMCP‐OG has an ACC of 0.65, SE of 0.689, SP of 0.618 and AUC of 0.703, which are 10%, 8.9%, 10.9% and 10.8% lower than those of DCAMCP, respectively. As a result, it is clear that combining two features can express the molecular structure more comprehensively than a single feature. In addition, DCAMCP‐NA has an ACC of 0.74, SE of 0.778, SP of 0.709 and AUC of 0.792. Compared with DCAMCP, its SE is the same as DCAMCP, but its ACC, SP and AUC are lower by 1%, 1.8% and 1.9%, respectively. Therefore, we believe that capsule network with self‐attention routing algorithms also bring improvements.

Moreover, we further analyse the individual components of DCAMCP based on the above results. First of all, we explore the impact of aggregation of various basic features. We argue that using a single type of feature to represent a molecule may not capture all its information. Basic molecular graph representations characterize only the atoms and do not identify some specific molecular substructures. Therefore, the results of using only the molecular graph as a feature are very unsatisfactory. The same problem exists when only fingerprint features are used. Because molecular fingerprints are usually abstract representations of specific molecular fragments, different molecules may have the same molecular fingerprints due to the same fragments, which will make it difficult for models to distinguish them. As a result, the combination of the two can better distinguish between different molecules, and DCAMCP could better capture those differences. Then, we preliminarily analyse the impact of the self‐attention routing capsule networks. As the capsule network operates on multidimensional vectors and this may contain more information than general scalar operations. For example, the modulus of the vector can represent the probability of the existence of the feature, the direction of the vector can represent the pose information of the feature,^35^ and its operation can represent the logical relationship between the vector components. This makes it possible for the capsule network to identify small changes in the vectors used to characterize molecules that can lead to dramatic changes in molecular properties. Simultaneously, DCAMCP successfully employs the self‐attention mechanism to improve the intrinsic ability of the capsule network, allowing vector information to be completely exploited and better predictions to be formed.

## 4 DISCUSSION AND CONCLUSION

In this work, we introduce a deep learning model based on capsule network and attention mechanism named DCAMCP to distinguish carcinogens from non‐carcinogens. We evaluate the predictive power and reliability of DCAMCP through fivefold CV with 100 repetitions and external validation. DCAMCP finally achieves an average ACC of 0.718 ± 0.009, SE of 0.721 ± 0.006, SP of 0.715 ± 0.014 and AUC of 0.793 ± 0.012. Furthermore, we use an independent external validation set to test DCAMCP, and the outstanding performance of DCAMCP on the external validation set demonstrates the robustness of DCAMCP in predicting carcinogens.

For the excellent predictive ability of DCAMCP, we believe that it may be attributed to the following points. First, more training samples are used in this study. Second, the molecular structure is more comprehensively and finely characterized by extracting and processing different molecular features. Third, the self‐attention routing capsule network has excellent performance in carcinogenicity prediction. In addition, it is not difficult to find from the performance of other models that traditional machine learning algorithms are more inclined to predict non‐carcinogens in the process of development, while deep learning algorithms show more comprehensively in the process of development.

However, DCAMCP still has several shortcomings. First, DCAMCP does not adequately represent carcinogenicity‐related substructures as previous methods. In addition, due to the continuous improvement of molecular fingerprints in recent years, the molecular fingerprints we use may be relatively lacking in expressive power. Besides that, because of the large number of hyperparameters, we lack exploration in the choice of hyperparameters for our model. Nonetheless, DCAMCP may still serve as a useful tool for filtering potential carcinogens in the early stages of drug discovery. The optimisation and improvement of DCAMCP deserves further exploration. We hope to improve the results of our model through better molecular characterisation methods such as EFCP fingerprints or mol2vec fingerprints, and effective hyperparameter optimisation algorithms such as evolutionary algorithms.

## AUTHOR CONTRIBUTIONS

**Zhe Chen:** Data curation (equal); investigation (equal); methodology (equal); writing – original draft (equal). **Li Zhang:** Conceptualization (equal); data curation (equal); investigation (equal); methodology (equal); writing – original draft (equal). **Jianqiang Sun:** Conceptualization (equal); formal analysis (equal); methodology (equal); supervision (equal). **Rui Meng:** Software (equal); validation (equal); writing – review and editing (equal). **Shuaidong Yin:** Software (equal); validation (equal); writing – review and editing (equal). **Qi Zhao:** Conceptualization (lead); funding acquisition (lead); methodology (lead); resources (lead); supervision (lead); writing – review and editing (lead).

## FUNDING INFORMATION

This study was supported by National Natural Science Foundation of China (Grant Nos. 11805091 and 82003655), Foundation of Education Department of Liaoning Province (Grant No. LJKZ0280), Shenyang Science and Technology Talent Project (Grant No. RC210216), Natural Science Foundation of Liaoning Province (Grant No. 2023‐MS‐288).

## CONFLICT OF INTEREST STATEMENT

The authors confirm that there are no conflicts of interest.

## Data Availability

The codes and datasets are available online at https://github.com/zhaoqi106/DCAMCP.
